# Visual perception in dyslexia is limited by sub-optimal scale selection

**DOI:** 10.1038/s41598-017-06967-6

**Published:** 2017-07-26

**Authors:** Richard Johnston, Nicola J. Pitchford, Neil W. Roach, Timothy Ledgeway

**Affiliations:** Visual Neuroscience Group, School of Psychology, The University of Nottingham, Nottingham, UK

## Abstract

Readers with dyslexia are purported to have a selective visual impairment but the underlying nature of the deficit remains elusive. Here, we used a combination of behavioural psychophysics and biologically-motivated computational modeling to investigate if this deficit extends to object segmentation, a process implicated in visual word form recognition. Thirty-eight adults with a wide range of reading abilities were shown random-dot displays spatially divided into horizontal segments. Adjacent segments contained either local motion signals in opposing directions or analogous static form cues depicting orthogonal orientations. Participants had to discriminate these segmented patterns from stimuli containing identical motion or form cues that were spatially intermingled. Results showed participants were unable to perform the motion or form task reliably when segment size was smaller than a spatial resolution (acuity) limit that was independent of reading skill. Coherence thresholds decreased as segment size increased, but for the motion task the rate of improvement was shallower for readers with dyslexia and the segment size where performance became asymptotic was larger. This suggests that segmentation is impaired in readers with dyslexia but only on tasks containing motion information. We interpret these findings within a novel framework in which the mechanisms underlying scale selection are impaired in developmental dyslexia.

## Introduction

Developmental dyslexia affects five to ten percent of the population and manifests as a difficulty with reading despite adequate tuition and educational opportunities^1^. The predominant view is that phonological awareness is impaired in developmental dyslexia^2^ but readers with dyslexia also have a selective visual impairment, which may impact on reading^3^.

Visual impairment in developmental dyslexia has been linked to vulnerability of dorsal stream processing^4^. Two anatomically distinct and functionally independent streams can be discerned in visual cortex, each specialised for encoding different types of information^5, 6^, but see ref. 7. The dorsal stream projects from primary visual cortex (V1) to parietal cortex and mediates global (overall) motion processing, spatial cognition and visual motor planning. The ventral stream projects from V1 to the temporal lobes and is implicated in global form (shape) perception, visual memory and object/face recognition. However, the independence of the dorsal and ventral streams has been questioned^8^.

To investigate the dorsal stream vulnerability hypothesis in adults with developmental dyslexia Hansen *et al* ^9^. devised a random-dot global motion task and a static global form task. The random-dot global motion task employed conventional random-dot kinematograms (RDKs) comprising a sequence of moving dots. Some dots moved in a common direction (*signal* dots), whilst others moved randomly (*noise* dots). Coherence thresholds, the minimum percentage of signal dots needed to reliably detect the global motion direction, were measured^10^. The global form stimuli consisted of static line segments. Some lines were orientated randomly, whilst others formed a concentric pattern. Readers with dyslexia had significantly higher coherence thresholds (by a factor of 2) than chronological age matched controls on the RDK task but not the global form task, consistent with the dorsal stream vulnerability hypothesis.

Some investigators have failed to uncover deficits in the processing of global motion, relative to global form in readers with dyslexia^11, 12^. Furthermore, recent work has cast doubt on whether global motion and global form tasks can be relied upon to clearly dissociate activity in the dorsal and ventral processing streams, as coherence thresholds on global motion and global form tasks are significantly and positively correlated^13, 14^. Hence, alternative explanations for the origin of visual impairment in dyslexia are needed. To explore if readers with dyslexia have a difficulty processing motion, temporal information, or integrating local visual cues across multiple (>2) dimensions Johnston *et al* ^13^. administered four, global motion and global form tasks to a large sample of adult readers. Two types of analyses were conducted. First, to investigate how general reading ability relates to task performance, a series of continuous analyses were conducted using a composite measure of reading skill in the entire sample. Next, to explore if the performance of individuals who have poor phonemic decoding skills, consistent with the dyslexic profile, differs to that of relatively good readers a series of between-group analyses were conducted. A similar pattern of results was found in both types of analyses. Generally poor readers and individuals with dyslexia exhibited relatively impaired performance on a conventional RDK task and a simpler global motion task comprising one-dimensional (1-D) bars. Crucially, both groups of poor readers had significantly higher coherence thresholds than relatively good readers on a temporally-defined global form task but not a static global form task. These results demonstrate that generally poor readers and individuals with dyslexia have difficulty processing temporal information, rather than motion *per se*.

An alternative explanation of the visual deficits in developmental dyslexia is the external noise exclusion hypothesis. In support of this hypothesis Sperling *et al* ^15^. found that readers with dyslexia had significantly higher contrast thresholds, than chronological age matched controls, when detecting static and flickering gratings embedded in high levels of external noise. However, this hypothesis fails to explain why readers with dyslexia do not always show impaired performance on visual tasks containing relatively high levels of external visual noise^13^. It is important to distinguish between external noise present in a visual stimulus and the internal noise inherent in the visual system. Recent work has shown that an increased level of internal noise does not limit visual perception in some neurodevelopmental disorders such as autism spectrum disorder^16^. There is some evidence to suggest that this might also be the case in readers with dyslexia^17, 18^ but this hypothesis has not yet been explored using motion and form tasks.

Another theory suggests that readers with dyslexia have difficulty forming a perceptual anchor to reduce perceptual memory load when discriminating between pairs of sequentially presented stimuli^19^. This theory, developed using auditory tasks, can also be applied to vision. To investigate the anchoring-deficit hypothesis Ahissar *et al* ^20^. employed a two-tone discrimination task in which participants identified the higher frequency tone. In the standard condition, a reference tone was presented at a fixed frequency, whilst in the no-standard condition the reference tone spanned a broad range. Thus, a perceptual anchor could act as a prior in the standard condition, reducing the load of online retention, whereas in the no-standard condition this was not possible because uncertainty existed regarding the frequency of the reference tone. Readers with dyslexia performed significantly worse than chronological age matched controls but only in the standard condition. This finding suggests that sensory deficits in developmental dyslexia are task- rather than stimulus-dependent. Whilst the anchoring-deficit hypothesis has received support^21^, it cannot readily explain selective impairments on visual tasks with low perceptual memory load i.e. when a single stimulus is presented on each trial^13^.

Another theory of the visual impairment in developmental dyslexia is the modified receptive field hypothesis^22^. It proposes that letter strings are processed in parallel by specialised detectors. The spatial extent or “receptive field size” of these detectors is thought to decrease during reading acquisition. If this is the case, one might expect visual crowding to be more pronounced in readers with dyslexia than relatively good readers but results are mixed^23, 24^. The modified receptive field hypothesis assumes that letter and shape stimuli are processed differently in V1, despite no evidence to support this. In addition, increased visual crowding has been reported in readers with dyslexia using a range of visual stimuli, not just letters^23^.

To date, all theories of the visual deficits in developmental dyslexia are challenged by recent research, suggesting a new framework is needed. To parse the visual scene into meaningful entities, such as text into words, or words into letters, the visual system must segment local features arising from different objects^25^. Computational models of reading suggest that this process mediates visual word form recognition^26–28^ and evidence suggests that the visual deficit in developmental dyslexia may compromise segmentation. To investigate this, Cornelissen *et al* ^29^. used a random-dot pattern, spatially segregated into three horizontal segments (each subtending 0.48°) by constraining dots in adjacent segments to move in opposing directions (leftwards and rightwards). Participants discriminated this stimulus from a uniform pattern containing dots moving in a common direction. Readers with dyslexia had significantly higher coherence thresholds (by a factor of 1.3) than controls matched for chronological age. However if participants based their decision on each trial by identifying the uniform pattern, then poorer performance for readers with dyslexia could just reflect the known difficulty in processing global motion (i.e. integration) rather than segmentation. Moreover a single measurement of sensitivity at a fixed segment size cannot fully characterise performance on object segmentation tasks, as performance depends upon segment size^30–32^.

In summary, mounting evidence suggests that readers with dyslexia have difficulty on visual tasks requiring *integration* of temporal information^13^. However, it is unclear if the perceptual deficit in developmental dyslexia extends to object *segmentation*. Previous research confounded integration with segmentation and relied on a single measurement of sensitivity at a fixed segment size^29^. We sought to address these issues by administering a motion task and an analogous form task, designed to measure object segmentation, to adults whose reading ability ranged along a continuum. Moreover, thresholds were measured for each of a range of segment sizes to investigate different components underpinning task performance.

## Materials and Methods

### Participants

Thirty-eight adults (15 Male, 23 Female) whose reading abilities ranged along a continuum were recruited either via a research participation scheme or Students Services at the University of Nottingham. The latter was important in order to obtain sufficient participants with reading difficulties. Mean age was 23.6 years (SD ± 36 months). All participants had English as their first language and were excluded if they had a neurodevelopmental disorder other than developmental dyslexia or ocular ill health. As individuals born prematurely typically have elevated global motion thresholds^33^, participants born <32 weeks gestation were excluded. All participants had normal or corrected-to-normal visual acuity and gave informed consent to take part in the study according to the Declaration of Helsinki. All experiments were performed in accordance with relevant guidelines and regulations. The ethics committee at the School of Psychology, University of Nottingham, granted ethical approval for the study.

### Psychometric tests

Non-verbal intelligence (IQ) was assessed using Raven’s Standard Progressive Matrices (SPM)^34^. Three measures of reading ability assessed different components of reading skill. The National Adult Reading Test (NART)^35^ consisting of 50 low-frequency irregular words was administered to measure whole-word lexical processing. To assess automaticity of reading aloud the Test of Word Reading Efficiency (TOWRE) Sight Word Efficiency subtest^36^ was used, comprising speeded reading of 104 regular words varying in frequency. The TOWRE Phonemic Decoding Efficiency subtest assessed sublexical decoding skills, measuring speeded reading of 63 pseudo-words varying in complexity. In both TOWRE subtests, participants were given 45 seconds to read as many words as possible, whereas the NART was self-paced. The dependent variable for each reading test was the number of words read correctly. Summary statistics characterising the reading abilities of the sample are shown in Table 1. A pertinent, defining feature of developmental dyslexia is poor phonemic decoding skills^2^. It is important to note that 16 participants (42% of the sample) had standard scores ≤85 (at or below the 15^th^ percentile) on the TOWRE Phonemic Decoding Efficiency subtest, which falls into the conventional range for diagnosing dyslexia^13, 37, 38^.

**Table 1:** Psychometric statistics for the entire sample.

	Mean	Standard Deviation	Range
NART (raw score/50)	26.97	6.57	7–39
TOWRE Sight Word Efficiency	85.63	12.58	69–113
TOWRE Phonemic Decoding	92.63	15.45	62–120
SPM (raw score/60)	51.18	5.48	39–59

Standard scores (*M* = 100, *SD* = 15) are shown unless otherwise stated. NART = National Adult Reading Test; TOWRE = Test of Word Reading Efficiency; SPM = Raven’s Standard Progressive Matrices.

### Visual stimuli

Stimuli were generated using MATLAB (MathWorks) and PsychToolBox^39^, and displayed on an Intergraph Interview 24hd96 monitor (refresh rate 100 Hz), which was carefully gamma-corrected. Stimuli were viewed binocularly (viewing distance 60 cm) and presented within a central 7 × 7° display window. Each stimulus was composed of “black” dots (diameter 0.07°) on a uniform “grey” (34 cd/m^2^) background. The stimulus duration was 0.43 s.

### Motion task

**Figure 1:**
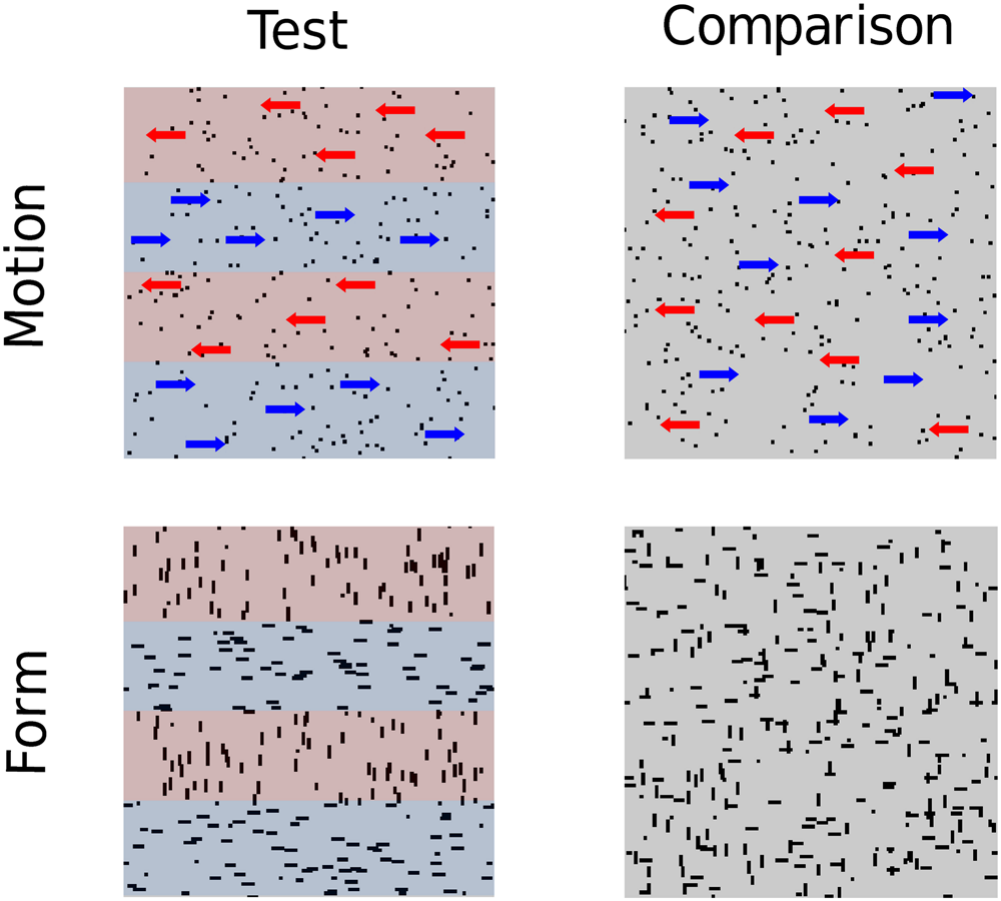
Schematic of the stimuli in the motion and form tasks. Coloured overlays and arrows have been added for illustrative purposes only and depict how the test stimuli were spatially segmented.

Motion stimuli (Fig. 1) consisted of forty-three images, each containing 256 dots, presented consecutively at 100 Hz to create apparent motion. Dots were displaced 0.035° on each positional update, resulting in a speed of 3.5°/s. Two patterns were presented in succession on each trial (inter-stimulus-interval 0.52 s) in a random order. The *test* stimulus was spatially divided into horizontal segments by constraining dots in adjacent segments to move in opposing directions (leftwards or rightwards). On each trial, the vertical spatial position of the segment boundaries was jittered to make them unpredictable. The participants’ task was to discriminate the *test* stimulus from a *comparison* stimulus containing identical motion cues that were spatially intermingled. Each dot had a limited lifetime and at the beginning of the motion sequence was assigned a random ‘age’ between 1 and 22 frames. This ‘age’ parameter was incremented on each image update and when the limit of 22 was exceeded the dot was replotted randomly within the same segment. Coherence could be varied between 0 and 100% by constraining some dots (*signal* dots) to move in the same direction and others to move randomly (*noise* dots). The coherence of the test and comparison stimuli was identical on a given trial and the only difference was the spatial distribution of the dots.

### Form task

The form stimuli (Fig. 1) were generated by computing a 4-frame, random-dot motion sequence. The individual frames were then spatially superimposed to create a static image^40^. Some dots (*signal* dots) formed localised streaks, orientated (vertically or horizontally) along a common axis, whilst others (*noise* dots) formed random clusters. The length of each dot streak was 0.18°. Two patterns were randomly presented in succession on each trial (inter-stimulus-interval 0.52 s) with equal probability. The *test* stimulus was spatially divided into horizontal segments by constraining differently oriented dot streaks to fall in adjacent segments. The vertical spatial position of the segment boundaries was jittered on each trial. The participants’ task was to discriminate the *test* stimulus from a *comparison* stimulus containing exactly the same form cues but these were spatially intermingled. Coherence could be varied between 0 and 100% by changing the proportion of signal to noise dot streaks.

### Procedure

#### Spatial resolution limits

To determine the smallest segment size needed to perceive reliably a spatially segmented pattern, the spatial resolution (acuity) limit was measured. Coherence was held constant at 100% and segment size was varied on each trial using a two-interval, temporal forced-choice procedure and a 3-down 1-up adaptive staircase tracking 79% correct. The initial step size was 3.5° and decreased by half after each reversal. The staircase terminated after 16 reversals and the arithmetic mean of the last six reversals was the acuity limit from that staircase. The reported acuity limit for each participant corresponds to the mean of at least five staircases.

### Coherence thresholds

Coherence thresholds were obtained in a similar manner to acuity limits. However, segment size was held constant in each trial block. It ranged from 0.438 to 3.5° in equal logarithmic steps. Coherence was varied on each trial using a 3-down 1-up staircase tracking 79% correct. The initial step size was equal to the total number of elements in the display and decreased by half after each reversal. The staircase terminated after 16 reversals and the arithmetic mean of the last six reversals was taken as the coherence threshold from that staircase. The reported threshold for each participant at a given segment size corresponds to the mean of at least five staircases.

### Curve-fitting

**Figure 2:**
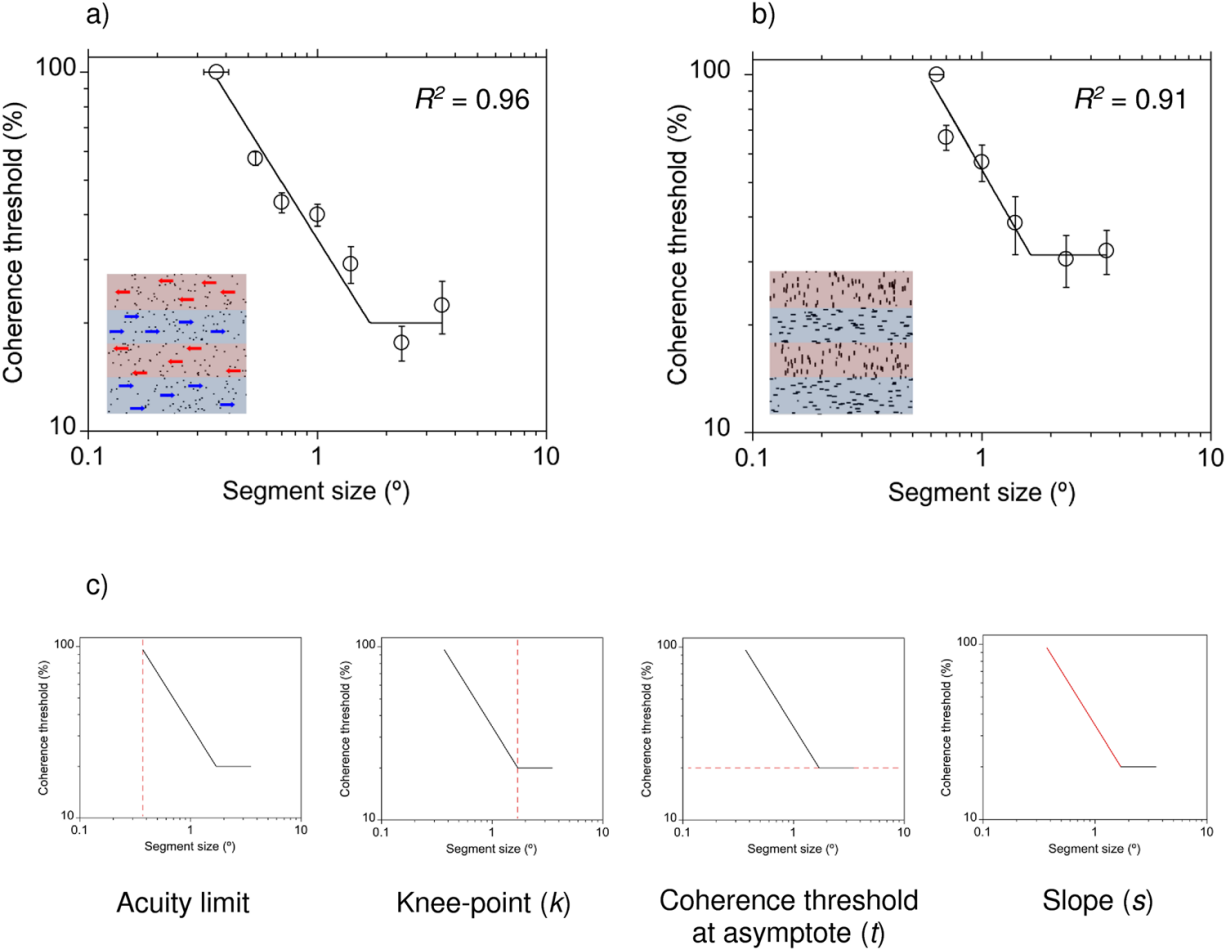
Data for a single participant on (**a**) the motion task and (**b**) the form task. The dashed red line in c) represents the spatial acuity limit and best-fitting parameters (*k*, *t*, & *s*) from Eq. 1. Error bars, ±1 sem.

To quantify the relationship between segment size and performance we fitted a two-limbed function to each participant’s data using a least-squares procedure. Data were well described (Mean *R* ^*2*^ for the motion task = 0.95, *SD* = 0.05, Range *=* 0.74 to 0.99; Mean *R* ^*2*^ for the form task = 0.94, *SD* = 0.04, Range *=* 0.83 to 0.99) by a function previously used to characterise performance on motion tasks^41, 42^

1 $$y=[\frac{({\rm{sgn}}(k-x)+1){(\frac{x}{k})}^{s}+{\rm{sgn}}(x-k)+1}{2}]t,$$

where *x* is segment size, and *k*, *t*, and *s* are constants. Parameter *k* is the knee-point of the function and represents the segment size above which performance no longer improves. Parameter *t* is the coherence threshold at asymptote, whilst parameter *s* is the slope of the descending limb of the curve (Fig. 2). Sgn(), the signum function, equals either −1, 0 or + 1 depending on whether the argument in parentheses is <0, 0 or >0, respectively. In four cases, the knee-point estimate (*k)* for the motion task exceeded the largest segment size (3.5°). For these cases, a conservative approach was taken and the curves were re-fitted with the knee-point parameter held constant at 3.5°.

### Statistical analyses

Dyslexia is primarily associated with poor phonemic decoding skills but the pattern of performance found on low-level visual perception tasks, requiring the processing of motion and form, cannot differentiate generally poor readers from individuals with poor phonemic decoding skills consistent with the dyslexic profile^13, 43, 44^. Preliminary analyses confirmed that this was also the case in the present study, so we took advantage of this finding and conducted a series of continuous analyses using a composite measure of reading skill. Adopting this type of experimental design enhances statistical power and means that arbitrary decisions about definitional criteria do not have to be made^45^. First, scores for the individual reading tests were z-transformed to allow comparison between different scores. Bi-variate correlations (Pearson’s product-moment correlation coefficient) were then used to investigate the relationships between the individual measures of reading ability in the entire sample. If correlations are strong, a composite score can be calculated by averaging z-scores for each reading test. However, if correlations are weak or moderate, principal component analysis (PCA) is more appropriate because it isolates a common construct that reflects a weighted average of the three individual measures of reading ability.

Gender and non-verbal IQ are sometimes associated with performance on global motion tasks^13, 46^. Therefore, semi-partial correlations were used to investigate if reading ability explained any additional variance. Acuity limits and curve-fit values for the motion and form task violated the assumption of normality, so correlations were assessed using Spearman’s rank-order correlation coefficient. However conducting parametric tests did not change the overall pattern of results. The Benjamini-Hochberg procedure^47^ was used to control for Type 1 errors when exploring the relationships between reading ability and different components underpinning task performance. This method is appropriate because only eight semi-partial correlations were conducted and it has greater statistical power, and is less prone to Type II errors, than simple Bonferroni correction^48^. A conservative false discovery rate of 0.05 was used to compute *p* values. Cohen’s *d* was calculated as a measure of effect size with 0.2 considered a small, 0.5 a medium, and 0.8 a large effect.

## Results

### Principal component analysis: Composite reading score

**Figure 3:**
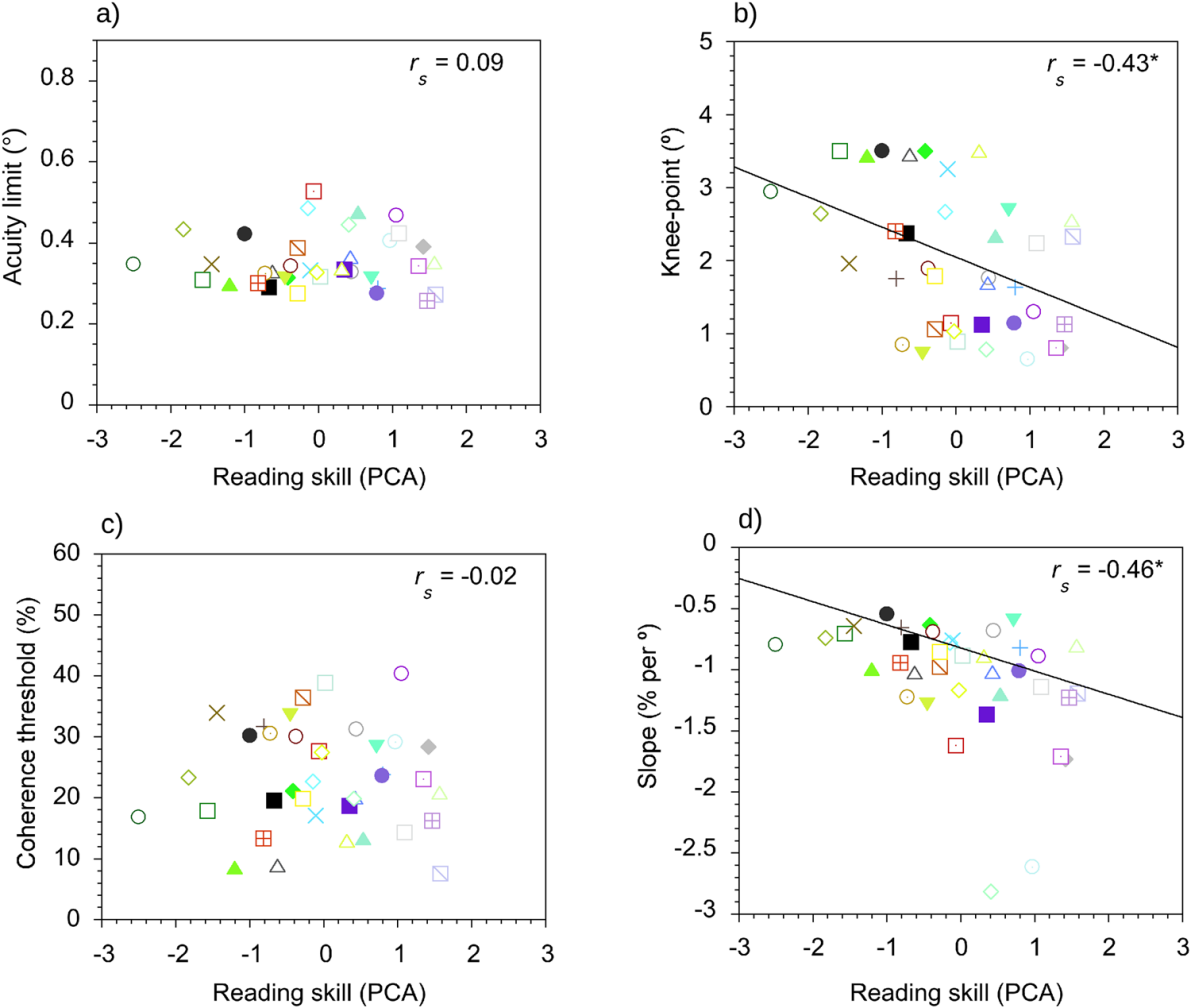
Scatter plots for the motion task in the entire sample of readers (*N* = 38) showing the relationships between reading ability and acuity limits (**a**), the knee-point of the curves (**b**), coherence thresholds at asymptote (**c**) and the slope of the descending limb (**d**). Each coloured symbol represents an individual participant. *p < 0.05; **p < 0.01; ***p < 0.001.

**Figure 4:**
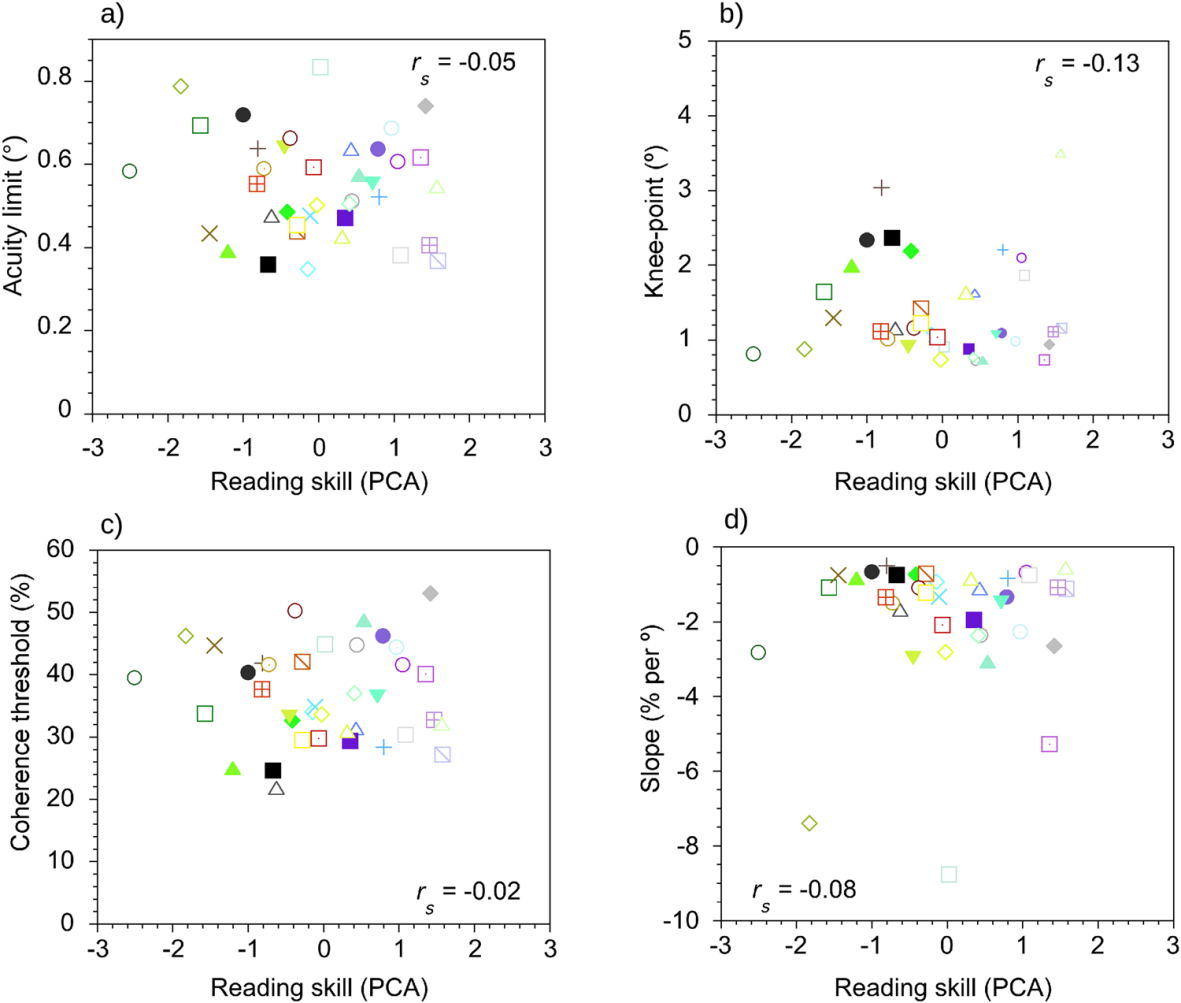
Same as Fig. 3 except the scatterplots show results for the form task.

To calculate the composite measure of reading skill PCA was performed as correlations between scores for the three reading tests were moderate to strong (*r* *=* 0.45 to 0.75, *p* *<* 0.01). Raw scores for the individual measures of reading ability were entered into the analysis, based on the correlation matrix. A single principal component accounted for 75% of the total variance amongst the reading tests (eigenvalue 1 = 2.26; eigenvalue 2 = 0.55; eigenvalue 3 = 0.19). Loadings for the NART and the TOWRE Sight Word Efficiency subtest were within the same range (0.81 and 0.85, respectively) but the TOWRE Phonemic Decoding subtest contributed more (loading = 0.94). PCA scores for each individual were entered into the whole-sample analyses to investigate how general reading ability relates to performance on the motion and form tasks (Figs 3 and 4).

### Semi-partial correlations: Motion task

The correlation between general reading skill and acuity limits on the motion task was not statistically significant, *r*_*s*_ = 0.09, *adjusted p-value* *=* 0.92, *d* = 0.18. However, there was a significant, negative correlation between reading ability and the knee-point of the curves, *r*_*s*_ = −0.43, *adjusted p-value* = 0.03, *d* *=* −0.95. The segment size at which performance became asymptotic was larger in generally poor readers (i.e. those with lower composite scores for reading) than relatively good readers (i.e. those with higher composite scores for reading). No significant correlation was found between reading skill and coherence thresholds at that asymptote, *r*_*s*_ = −0.02, *adjusted p-value* *=* 0.92, *d* = −0.04, but there was a significant, negative correlation between general reading ability and the slope of the descending limb of the curves, *r*_*s*_ = −0.46, *adjusted p-value* *=* 0.03, *d* = −1.04. The rate of improvement as segment size increased was shallower in generally poor readers than relatively good readers. Gender and Non-Verbal IQ were not significantly associated with acuity limits nor any of the curve-fit values.

### Semi-partial correlations: Form task

The correlation between the composite measure of reading skill and acuity limits on the form task was not statistically significant, *r*_*s*_ = −0.05, *adjusted p-value* *=* 0.92, *d* = −0.10. Furthermore, no significant correlation was found between reading ability and the knee-point of the curves, *r*_*s*_ = −0.13, *adjusted p-value* *=* 0.92, *d* = −0.26. Reading ability was not significantly correlated with coherence thresholds at asymptote, *r*_*s*_ = −0.02, *adjusted p-value* *=* 0.92, *d* = −0.04, nor the slope of the descending limb of the curves, *r*_*s*_ = −0.08, *adjusted p-value* = 0.92, *d* = −0.16. Gender and Non-verbal IQ were not significantly associated with acuity limits nor any of the curve-fit values for the form task.

### Computational analyses

To investigate why the poorest readers exhibited a different performance on the motion segmentation task computer simulations were performed. The test and comparison stimuli used in these analyses were identical to those in the psychophysical tasks except the motion-sequences comprised two frames for computational efficiency. Acuity limits and coherence thresholds were obtained using a 3-down 1-up staircase. They correspond to the mean of at least five staircases. Precise details of the computational analyses are provided below.

**Figure 5:**
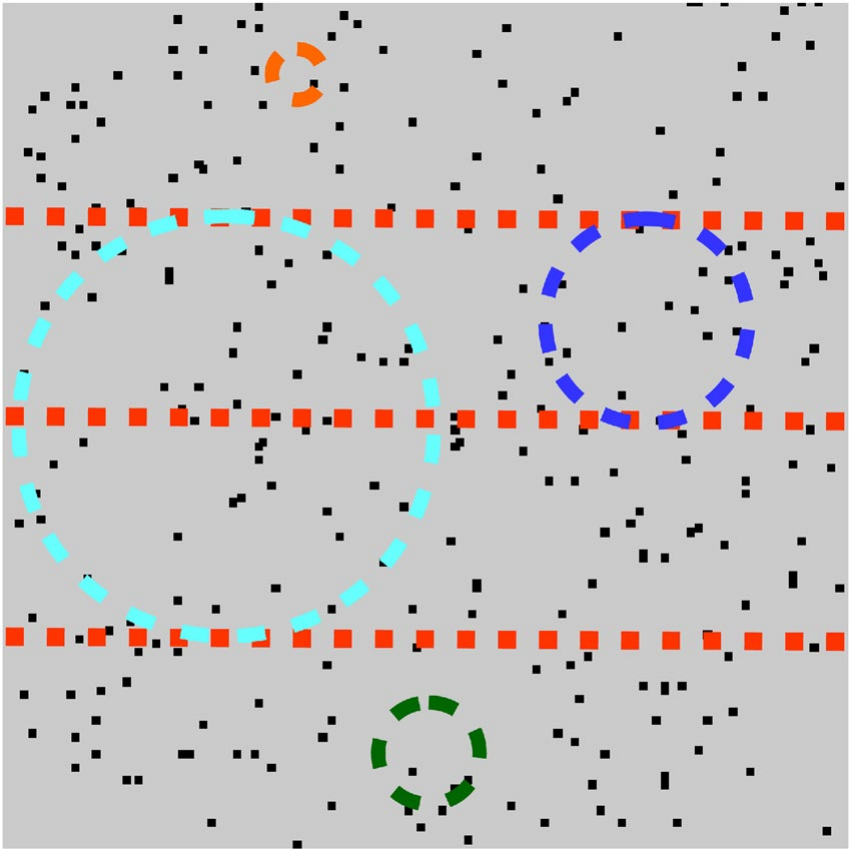
Schematic illustration of the computational analyses performed at a range of spatial scales. Circular integration fields of various sizes are shown superimposed upon a single frame of the spatially-segmented test stimulus, in which the dots underwent motion.

At each location (*i*, *j*) in the image the net opponent motion (rightwards minus leftwards) was calculated as the sum of the horizontal direction components of all the dots (*T*) contained within a circular integration field of variable diameter (Fig. 5) centred at that location:

2 $${R}_{ij}=\sum _{d=0}^{T}\,{\cos }\,{\theta }_{d}$$

where *R*_*ij*_ is the net opponent motion and *θ*_*d*_ is the direction of each dot. This type of analysis is biologically plausible as electrophysiology, human brain imaging and psychophysics support the existence of motion opponency in visual cortex^49–52^. Furthermore motion-sensitive neurons operate over a range of spatial scales and there is considerable scatter in the size of receptive fields available at each location in the visual field^53, 54^.

The directional variance across space was then computed for each motion sequence as:

3 $${\sigma }^{2}=\frac{1}{n}\sum {({R}_{mean}-{R}_{ij})}^{2}$$

where *R*_*mean*_ is the mean net opponent motion, averaged across all spatial locations (*n*) in the image.

A decision was then made on each trial, regarding the identity of the test stimulus, based on the motion sequence with the largest directional variance. If *σ* ^2^ for the test stimulus exceeded *σ* ^2^ for the comparison, the decision was correct. In the rare circumstance that the two variances were identical, the response was randomly assigned either correct or incorrect with equal probability.

**Figure 6:**
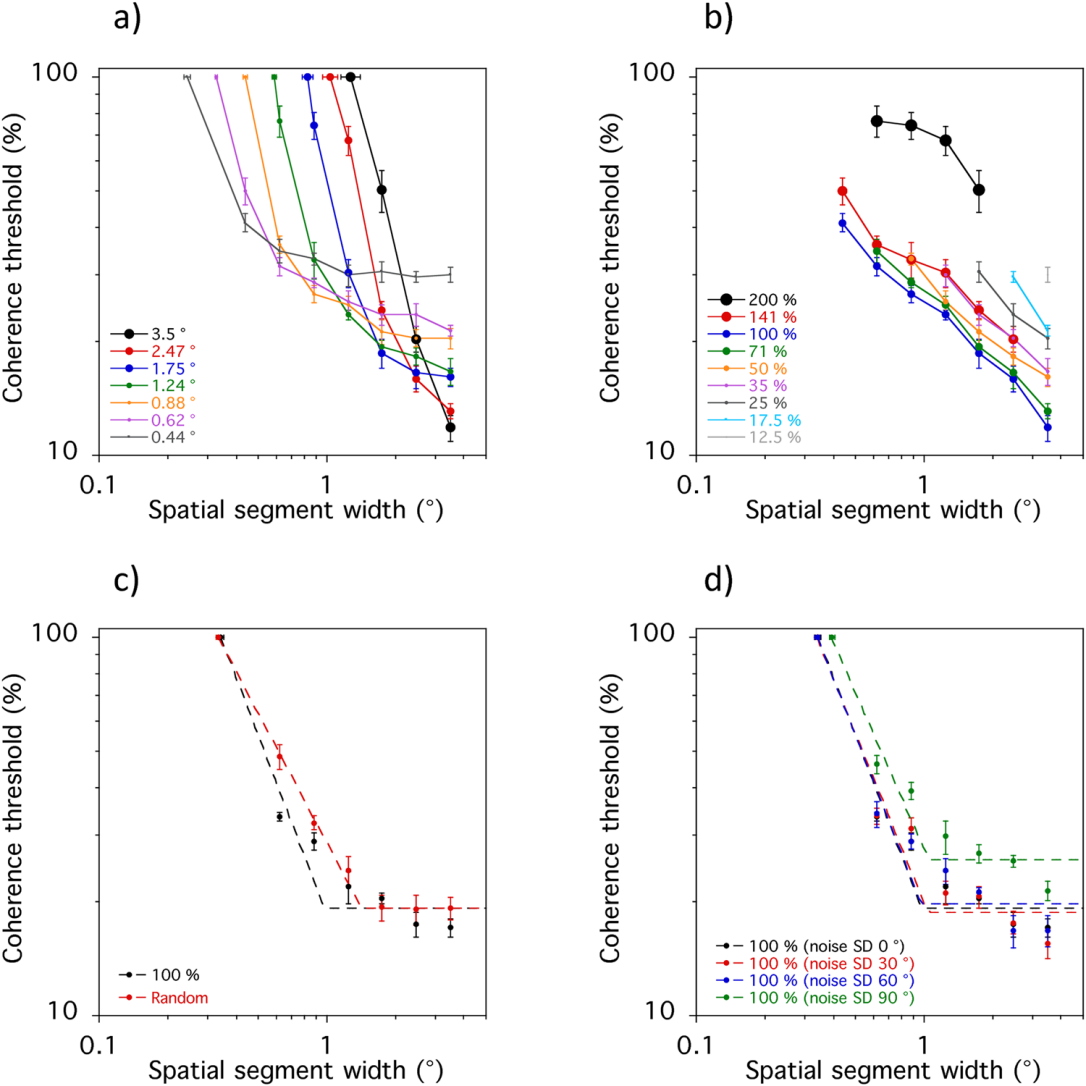
Results of the computational analyses. The graphs display acuity limits and coherence thresholds as a function of segment size when integration area is either fixed (**a**) or varied in proportion to segment size (**b**). Upper and lower bounds on field size were introduced to constrain the analyses and produced a qualitatively similar pattern of performance to that associated with poor reading when integration field size was randomised (sub-optimal) on each trial (**c**), but not when internal noise levels were elevated (**d**).

First, integration field size was varied between 0.438 and 3.5° (Fig. 6a). Acuity limits increased as integration field size increased. This resulted in a rightward shift of the curves and suggests that acuity limits represent the minimum integration field size that can be used. Secondly, coherence thresholds decreased as segment size increased, regardless of integration field size. There is evidence of asymptotic behaviour when the integration field size matched the segment size. For example, coherence thresholds became asymptotic at ~0.88° when the integration field size was 0.88°.

Next, integration field size was varied in proportion to segment size (Fig. 6b). Coherence thresholds were lowest when integration field size matched the segment size but performance worsened when it decreased by 25 or 50%. There was a dramatic increase in coherence thresholds when the integration field size was larger (e.g. 200%) than the segment size. These findings suggest that performance on the motion task is optimal when the size of the integration field closely matches the segment size. Overall sensitivity is greatly reduced when larger integration fields are employed to perform the task.

Nominal upper (1.24°) and lower (0.62°) bounds were then introduced to constrain the analyses. The upper bound corresponds to the maximum integration field size that can be used to perform the motion task, whilst the lower bound determines the acuity limit. When segment size increases beyond the upper bound coherence thresholds become asymptotic because a smaller, sub-optimal integration field has to be employed. Figure 6c shows that coherence thresholds decreased as segment size increased. The slope of the descending limb of the curve was much shallower when integration field size was chosen at random on each trial so that it did not always match the segment size. The knee-point at which coherence thresholds became asymptotic was also larger. These results are, to a first approximation, similar to those observed in readers with dyslexia and imply sub-optimal integration fields might be used to perform the motion task.

To explore if increased levels of internal noise could provide an alternative explanation for our results Gaussian noise was added to the model’s encoded direction of each dot (Fig. 6d). Integration field size was set to match the segment size (100%), whilst the standard deviation (SD) of the Gaussian noise distribution was varied between 0 and 90°. Adding a relatively small amount of internal noise (SD 30–60°) did not change the overall pattern of results, but the acuity limit and coherence threshold at asymptote increased dramatically when the standard deviation of the Gaussian noise was 90°. Crucially, there was no change in the slope of the descending limb or the knee-point of the curve. These findings are the opposite of those observed in the poorest readers and demonstrate that elevated levels of internal noise cannot readily explain their performance on the motion-based segmentation task.

## Discussion

The current study investigated if the perceptual deficit in developmental dyslexia extends to object *segmentation*. Thirty-eight adults with a wide range of reading abilities, of which 16 (42%) met the conventional criterion for developmental dyslexia^13, 37, 38^, were tested on motion- and form-based spatial segmentation tasks. Results showed that acuity limits were independent of reading ability, indicating that the minimum segment size needed to perform the motion and form tasks was normal in less-skilled readers. Reading ability was significantly associated with performance but crucially only for the motion task, such that the segment size at which performance became asymptotic (i.e. the knee-point) was larger in the poorest readers and resulted in a flattening of the descending limb of the curves. Importantly these results demonstrate that the perceptual deficit in developmental dyslexia extends to object segmentation, but only on tasks containing motion information.

Our results suggest that overall sensitivity on the segmentation tasks was normal in readers with dyslexia, as coherence thresholds at asymptote were not significantly associated with reading ability. This might seem at odds with the results of Cornelissen *et al*.^29^ as they reported that readers with dyslexia had significantly higher coherence thresholds on a similar motion-based segmentation task. However, segment size was fixed at 0.48° in their study and represents a single point along the descending limb of the function (Fig. 2a). The poorest readers in our sample had higher coherence thresholds than good readers at this point too, highlighting the importance of not relying on a single measurement of sensitivity to characterise performance.

Our previous work challenged the dorsal stream vulnerability hypothesis as an explanation for visual deficits in developmental dyslexia^13^. Similarly recent research has cast further doubt on the putative independence of the dorsal and ventral streams as coherence thresholds on RDK tasks and static global form tasks are significantly and positively correlated^13, 14^. In addition, it is unclear if the dorsal pathway is actually involved in the segmentation of motion cues, as cells in the ventral stream (e.g. area V4) of primates respond to kinetic boundary stimuli similar to those used in the present study^55^. The noise exclusion hypothesis^15^, the anchoring-deficit hypothesis^19^ and the modified receptive field hypothesis^22^ are also unsupported by the current study as they all predict that readers with dyslexia should show impaired performance on the form task as well as the motion task, but this was not the case.

Thus, our findings are problematic for current theoretical frameworks. The rate of improvement for motion-based segmentation was shallower in readers with dyslexia and the segment size at which coherence thresholds reached asymptote was larger. In light of the computational simulations, we argue that this pattern of impairment arises because of a deficit with scale selection. It is unlikely to reflect increased levels of internal noise because exactly the opposite pattern of results would have been expected (Fig. 6d). A difficulty with scale selection could impact on the reading process because object segmentation plays a major role in orthographic processing^26–28^. Such a deficit could either be the cause or a consequence of dyslexia and this could be explored in the future using longitudinal or training studies^56^.

An important issue concerns why readers with dyslexia have difficulties selecting optimally sized integration fields on the motion task but not the form task. During reading characteristic saccadic eye movements and fixations are made^57^, changing the position of words on the retinae over time. The motion task in the current study also contained time-varying information, whereas the visual cues in the form task remained relatively static throughout the stimulus presentation as participants were required to maintain steady fixation in the centre of the stimulus. Thus, readers with dyslexia might have a deficit with scale selection on visual tasks that require segmentation of a temporally-changing input. Whether or not this can explain why performance on conventional RDKs is impaired in developmental dyslexia, remains unresolved. RDKs require spatio-temporal integration and for typically developing adults thresholds improve when stimulus size increases, presumably because more visual information becomes available^58, 59^. If readers with dyslexia select (say) smaller integration fields than skilled readers, it may explain why they also have elevated coherence thresholds for RDKs^3^.

In summary, the perceptual deficit in developmental dyslexia extends to object segmentation but only on tasks containing motion information. These findings support a novel framework, in which the mechanisms underlying scale selection are impaired in adults with developmental dyslexia. We have argued that this could either be the cause or a consequence of difficulties recognising visual word forms. During reading the position of words on the retinae changes over time, which could explain why readers with dyslexia have difficulty with scale selection on the motion task but not the form task^60^.
